# Supplementary material for: Host Resistance, Genomics and Population Dynamics in a Salmonella Enteritidis and Phage System
Source: Viruses. 2019 Feb 22;11(2):188. doi: 10.3390/v11020188 (PMC6410252; doi:10.3390/v11020188)
Supplement: Supplementary file 1 [file viruses-11-00188-s001.pdf]

Supplementary Materials:

1. Basic Reproduction Number and Proliferation/Inundation Thresh- old Analysis

The probability of a single phage successfully infecting a susceptible bacterium can be calculated from Equation 2 as $\beta_1 S$. The probability $P(i)$ of a phage doing so before being degraded due to free phage decay at rate μ_1 is given in Equation S7.

$$P(i) = \frac{\beta_1 S}{\beta_1 S + \mu_1}$$

(S7)

The Basic Reproduction Number $\mathcal{R}_0$ for a phage in the systems described by Equation 2 can now be calculated from the definition of the term as the average number of new free virions produced by a single free phage in a population of bacteria that is susceptible to it (1). As shown in Equation S8, the average new phages produced by a single free phage is equal to the number of phages produced by a successful infection event, given by the burst size b_1, times the probability $P(i)$

$$\mathscr{R}_0 = \frac{\beta_1 S}{\beta_1 S + \mu_1} b_1$$

(S8)

For the two-phage case in Equation 1, the expression is modified for each phage to include the population susceptible specifically to the given phage, as shown in Equation S9.

$$\mathscr{R}_{0,1} = \frac{\beta_1 (S + R_2)}{\beta_1 (S + R_2) + \mu_1} b_1$$
$$\mathscr{R}_{0,2} = \frac{\beta_2 (S + R_1)}{\beta_2 (S + R_1) + \mu_2} b_2$$

(S9)

As has already mentioned, by definition of $\mathcal{R}_0$, when $\mathcal{R}_0 < 1$, the phage population will decrease, whereas if $\mathcal{R}_0 > 1$, the phage population increases. Therefore, the size of the population of bacteria susceptible to a given phage when $\mathcal{R}_0 = 1$ defines the proliferation threshold. Equation S9 can be rewritten into the form shown in Equation S10.

$$S + R_2 = \frac{\mu_1}{\beta_1 (b_1 - 1)}$$
$$S + R_1 = \frac{\mu_2}{\beta_2 (b_2 - 1)}$$

(S10)

Equation S10 establishes the proliferation thresholds for Phages 1 and 2 shown in Equation 3, in terms of the total sum of bacteria susceptible to a given phage.

The inundation threshold is defined as the phage population at which the bacterial population susceptible to it starts to decline (2). In the system described by Equation 2, this is equivalent to the phage population $V_{I,1}$ at which $dS/dt = 0$. Solving the equation for dS/dt in the system described by Equation 2 in terms of V_1 at steady state yields the expression in Equation S11.)

$$V_{I,1} = \frac{r(1 - a\alpha)\left(1 - \frac{N}{k}\right) + m\left(\frac{R_1}{S} - 1\right)}{\beta_1}$$

(S11)

In the two-phage case described in Equation 1, the susceptible and resistant populations specifically for each phage are taken, and the inundation thresholds are as shown by in Equation S12.

$$V_{I,1} = \frac{r(1-a\alpha)\left(1-\frac{N}{k}\right)+m\left(\frac{R_1+R_{1,2}}{R_2+S}-1\right)}{\beta_1}$$
$$V_{I,2} = \frac{r(1-a\alpha)\left(1-\frac{N}{k}\right)+m\left(\frac{R_2+R_{1,2}}{R_1+S}-1\right)}{\beta_2}$$

(S12)

These expressions describe the inundation threshold at all times in the system. However, it is desirable to obtain expressions in terms of the system and phage-specific parameters that allow the comparison of different phages. To do this, an upper bound for the inundation threshold can be established by assuming exponential growth, as shown in the inequality in Equation S13.

$$V_{I,1} < \frac{r(1-a\alpha)+m\left(\frac{R_1+R_{1,2}}{R_2+S}-1\right)}{\beta_1}$$
$$V_{I,2} < \frac{r(1-a\alpha)+m\left(\frac{R_2+R_{1,2}}{R_1+S}-1\right)}{\beta_2}$$

(S13)

Finally, it is possible to only consider the case leading up to the first peak of infection for each phage. In this situation in the model, the number of cells susceptible to a given phage far exceeds the number of resistant cells, due to the model's starting conditions with a completely susceptible population and the fact that $r >> m$. Thus, it is possible to define upper bounds for the inundation thresholds of Phages 1 and 2 in the context of the first infection peak, as shown in Equation S14.

$$V_{IP,1} < \frac{r(1-a\alpha)-m}{\beta_1}$$
$$V_{IP,2} < \frac{r(1-a\alpha)-m}{\beta_2}$$

(S14)

Equation S14 establishes the inundation thresholds bounds for Phages 1 and 2 shown in Equation 4, in terms of the total sum of bacteria susceptible to a given phage. In the case of single phage populations without antibiotic, the expressions for the thresholds found in Equations S10 and S14 are the same as those found by Cairns et al. (2). The proliferation threshold is equivalent to that found by Payne & Jansen (3,4) if mutation is not taken into account.

Supplemental Figures

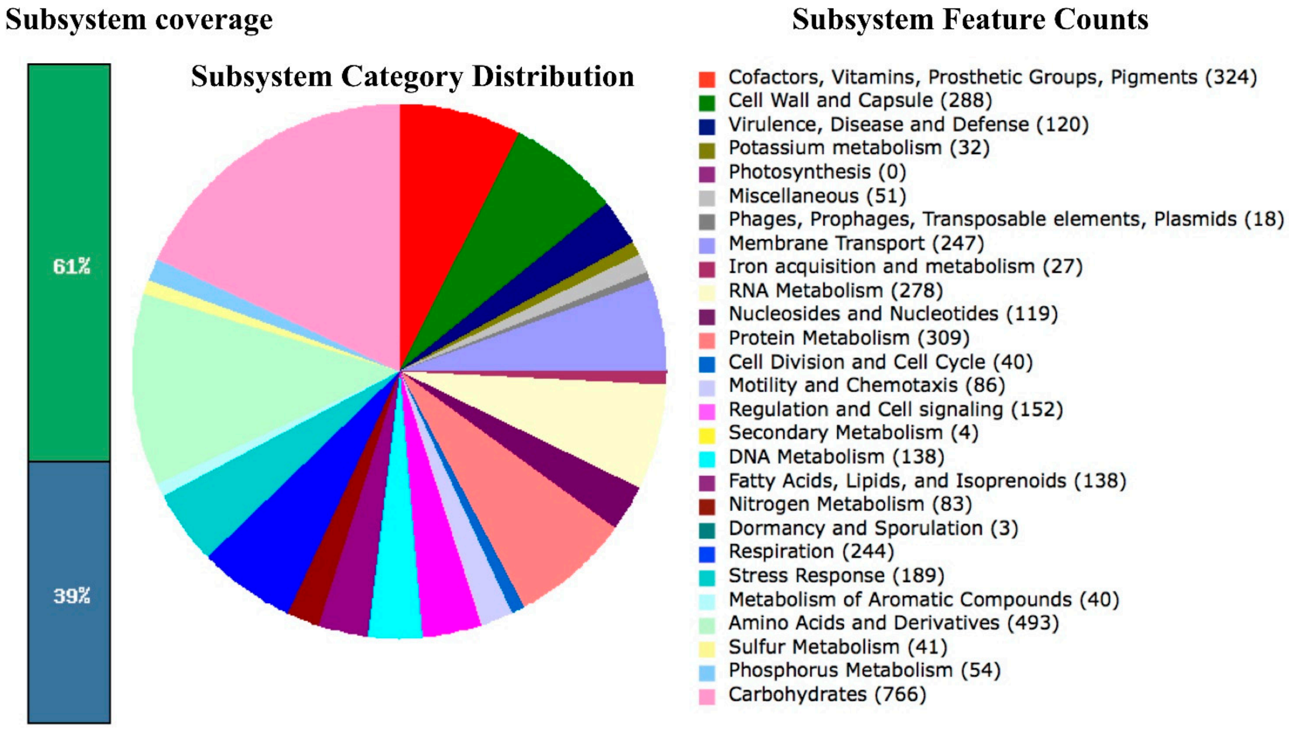

Figure S1. Salmonella Enteritidis s25pp Gene annotation.

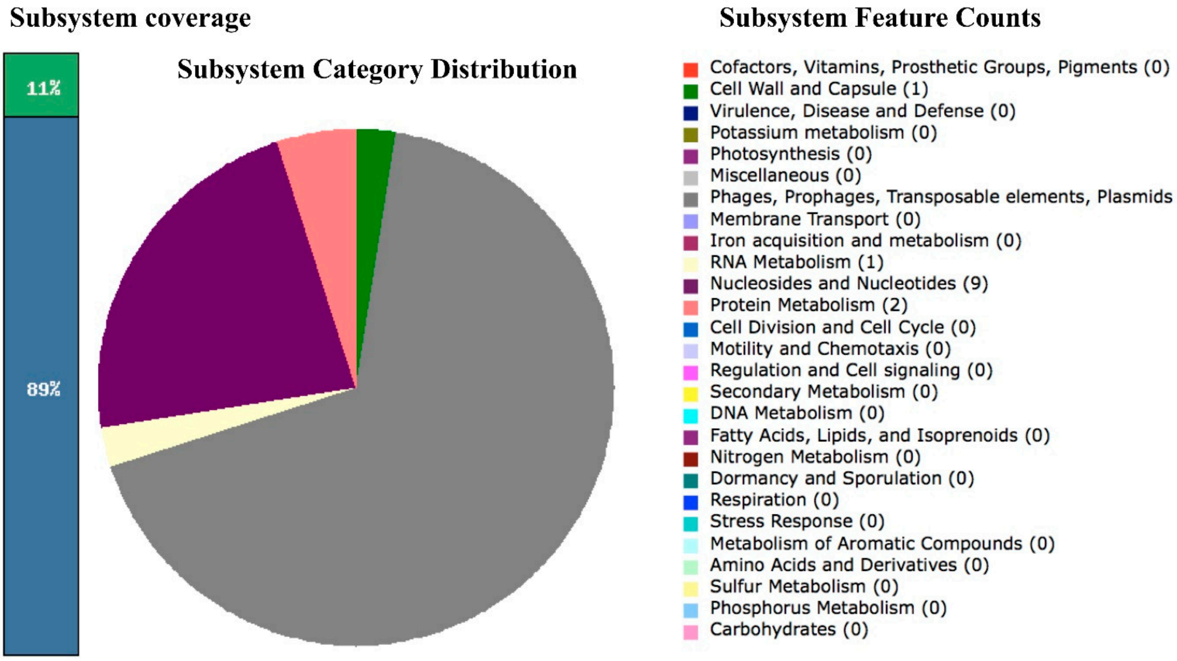

Figure S2. Bacteriophage φSan23 gene annotation.

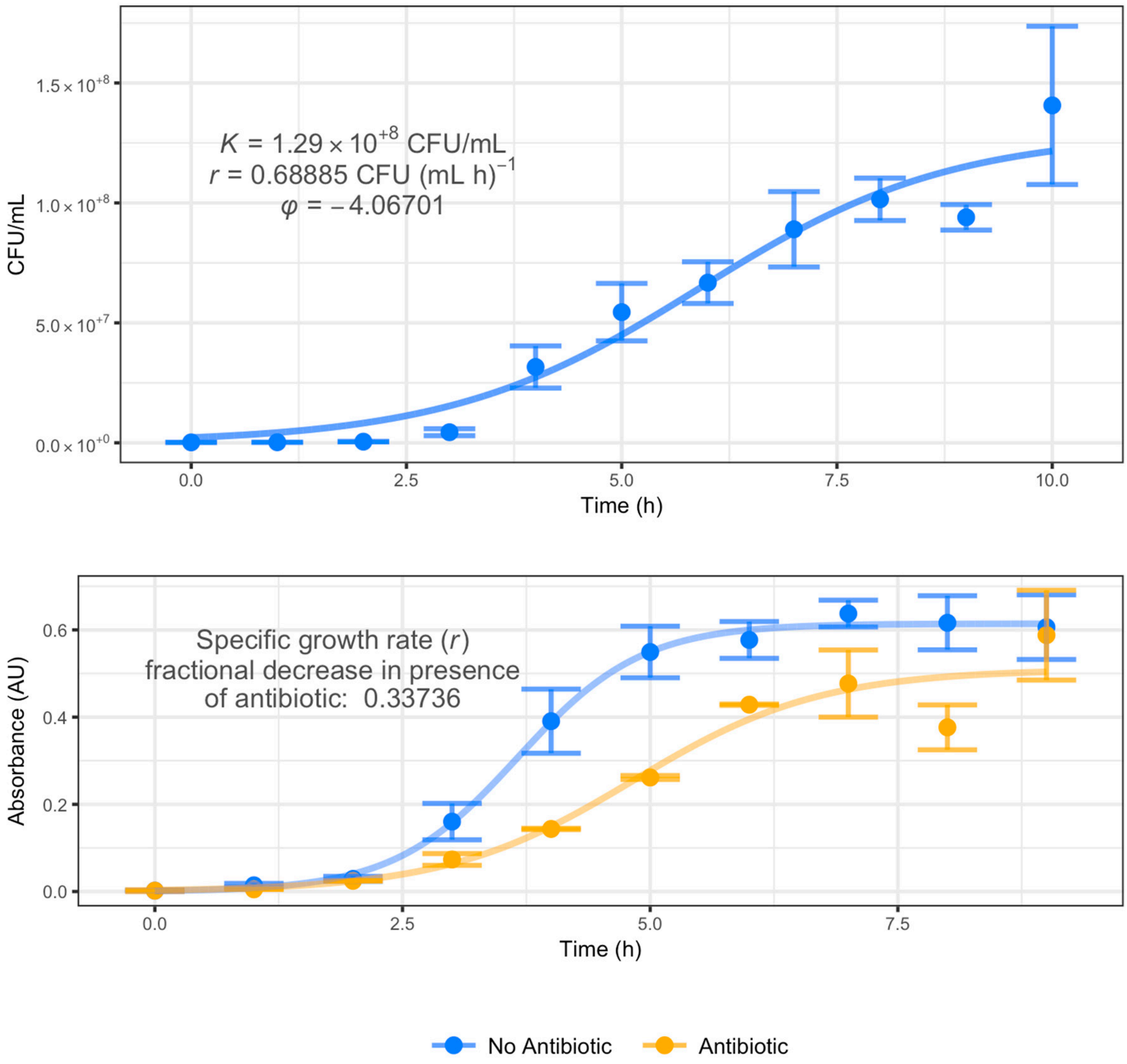

Figure S3. Nonlinear fit of growth curve data with logistic function used for determining logistic growth parameters of model. Error bars portray standard error ($n = 3$).

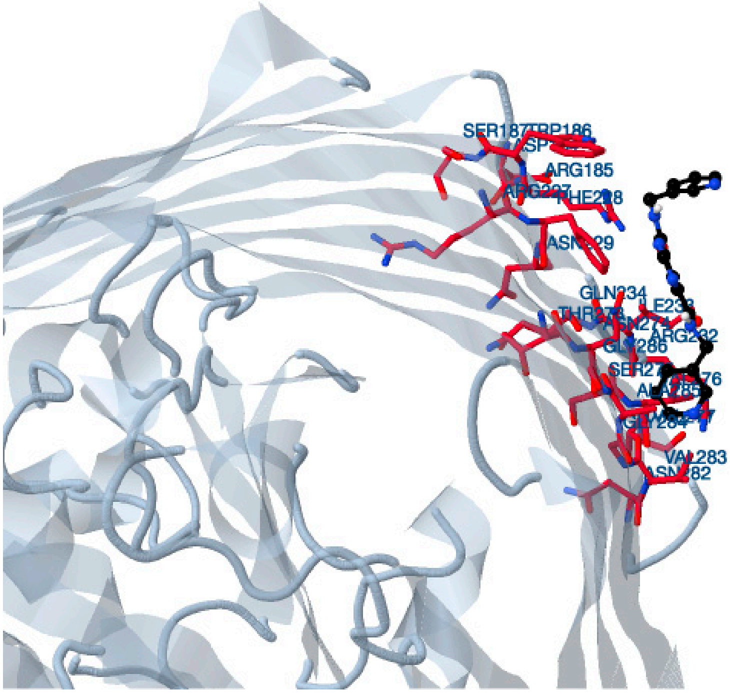

Figure S4. Molecular docking between BtuB receptor and φSan23 tail protein.

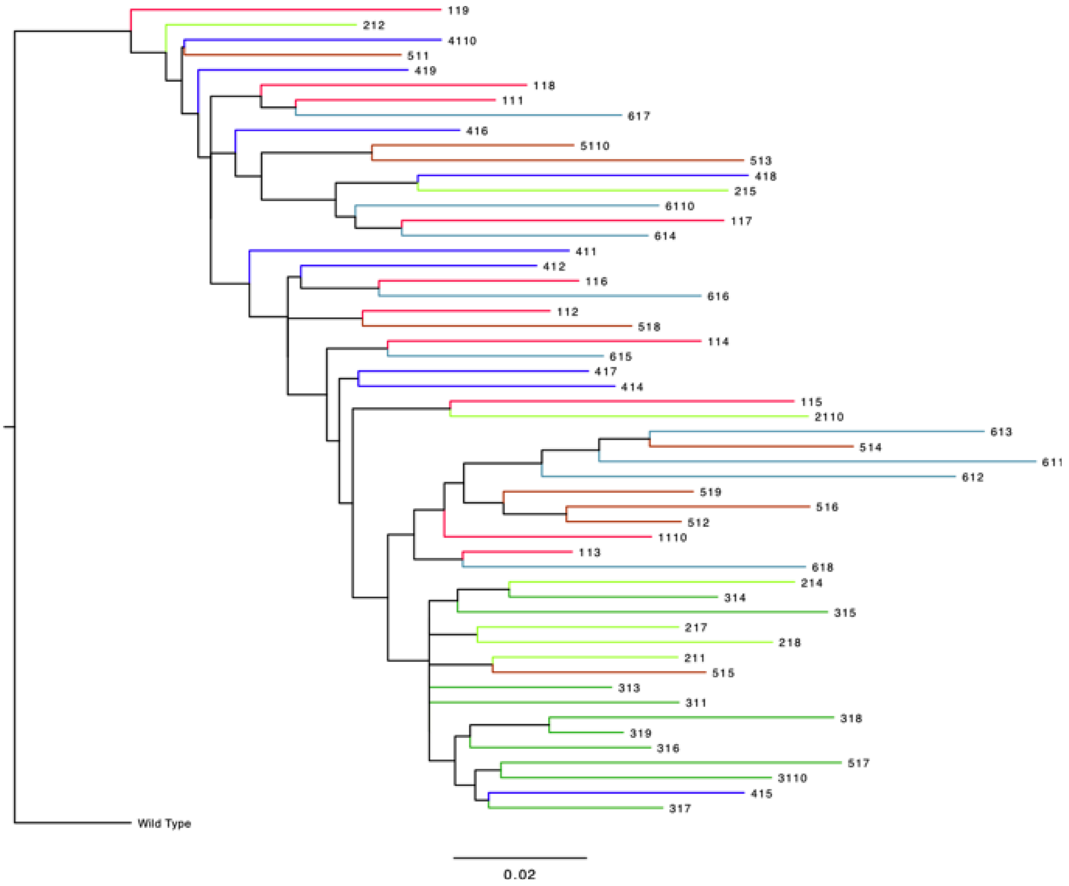

Figure S5. SNP tree based on SNP calling performed from mutants over time. Each color represents transfer number: transfer 1 is red, transfer 2 is fuchsia, transfer 3 green, transfer 4 is blue, transfer 5 is black, and transfer 6 is orange. SNPs did not show any pattern that could be associated to the transfer.

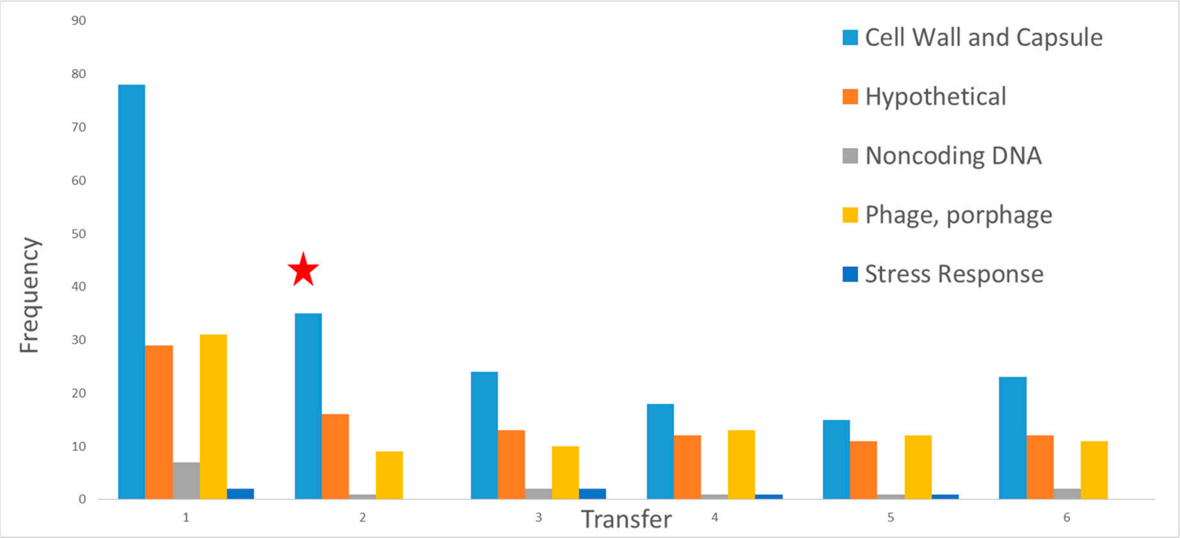

Figure S6. Shows the distribution of the snps along each transfer and with the category determined.

Supplemental Video 1

https://avholguin103.wixsite.com/coevolution

Works cited

1. Brauer F, Castillo-Chavez C, Castillo-Chavez C. 2001. Mathematical models in population biology and epidemiology. Springer.

2. Cairns BJ, Timms AR, Jansen VAA, Connerton IF, Payne RJH. 2009. Quantitative models of in vitro bacteriophage--host dynamics and their application to phage therapy. PLoS Pathog 5:e1000253.

3. Payne RJH, Jansen VAA. 2000. Phage therapy: the peculiar kinetics of self-replicating pharmaceuticals. Clin Pharmacol Ther 68:225–230.

4. Payne RJH, Jansen VAA. 2003. Pharmacokinetic principles of bacteriophage therapy. Clin Pharmacokinet 42:315–325.
